# Frequency, diagnosis, and management of polymyalgia rheumatica in Germany—database analysis of medical insurance data

**DOI:** 10.1093/rheumatology/keaf367

**Published:** 2025-07-07

**Authors:** Wolfgang A Schmidt, Marco Alibone, Paul Ludwig, Dominik Obermüller, Franziska Karl, Stephanie Terner, Nils Venhoff

**Affiliations:** Rheumatology, Waldfriede Hospital, Berlin, Germany; InGef-Institute for Applied Health Research Berlin GmbH, Berlin, Germany; InGef-Institute for Applied Health Research Berlin GmbH, Berlin, Germany; InGef-Institute for Applied Health Research Berlin GmbH, Berlin, Germany; Novartis Pharma GmbH, Nürnberg, Germany; Novartis Pharma GmbH, Nürnberg, Germany; Internal Medicine, Department of Rheumatology and Clinical Immunology, Medical Center—University of Freiburg, Freiburg, Germany

**Keywords:** polymyalgia rheumatica, incidence, prevalence, medical insurance data, comorbidities, glucocorticoids, methotrexate

## Abstract

**Objectives:**

Epidemiological data on polymyalgia rheumatica (PMR) in Germany is limited. Current national prevalence estimates are low by international standards. This study aimed to gain up-to-date data representative of Germany.

**Methods:**

A cross-sectional analysis was conducted on a sample of 4.8 million insured individuals, representative of the German population, from the InGef (Institute for Health Research Berlin GmbH) research database. Inclusion criteria were age ≥50 years, continuous insurance status for a base period of 3 years and for the subsequent 2 years for longitudinal analysis. Results were additionally extrapolated to the German population.

**Results:**

Each year from 2018 to 2021, around 1.7 million insured individuals were included in the study. Extrapolated to the German population in 2021, the incidence was 111/100 000 and the prevalence was 937/100 000. Diagnosis was made predominantly in outpatient settings (86.7%) most frequently by general practitioners (GP; 37.1%), internists (22.2%), rheumatologists (11.4%) and orthopaedists (10.2%). An additional 21% were referred to rheumatologists for treatment after diagnosis. Treatment was most commonly initiated by GPs, followed by rheumatologists, and included methotrexate in 19.8%. Most common comorbidities of prevalent patients comprised arterial hypertension (75.9%), dyslipidaemia (55.0%), diabetes mellitus (29.7%), osteoporosis (27.1%), coronary heart disease (23.4%) and cataract (24.3%).

**Conclusion:**

This analysis revealed that PMR occurs more frequently in individuals aged 50 or older in Germany than previously assumed. Diagnosis is primarily made in general practice settings, with about one-third of patients being treated by rheumatologists. Comorbidities such as diabetes mellitus or cardiovascular diseases are common in the prevalent population.

Rheumatology key messages

- Incidence and prevalence of PMR in Germany are higher than previously estimated.
- Rheumatologists treat about one-third of the patients.
- Comorbidities are common in the prevalent population.

## Introduction

Polymyalgia rheumatica (PMR) is the second most frequent inflammatory immune-mediated disease following rheumatoid arthritis in people over the age of 50 years [1, 2]. Women are more likely to be affected by PMR [3]. The symptoms of PMR include severe proximal muscle pain and morning stiffness, especially in the shoulder and neck, and, less frequently, in the pelvic girdle [3, 4]. Fatigue, arthralgia, weight loss, anorexia and low grade fever are common [3].

Provisional classification criteria of the European Alliance of Associations for Rheumatology (EULAR) and the American College of Rheumatology (ACR) include age ≥50 years, increased inflammatory markers, shoulder and pelvic girdle pain, morning stiffness, negative rheumatoid factor and anti-citrullinated antibodies, and conspicuous findings on shoulder and hip ultrasound [5]. The diagnosis of PMR should be based on typical clinical signs and symptoms, and exclusion of other diseases, such as rheumatoid arthritis, shoulder and hip osteoarthritis, and calcium pyrophosphate deposition disease [3, 6]. However, diagnosis of PMR remains challenging due to the non-specific or generic symptoms as well as the heterogeneous presentation and course of disease [4].

Particularly proximal muscle pain and stiffness in PMR are linked to an inflammatory immune response [7]. About 30% of PMR cases are associated with giant cell arteritis (GCA), a chronic inflammatory vasculitis with similar demographic features [3, 7]. Both disorders show elevated IL-6 levels and an imbalance between proinflammatory T-helper 17 (Th17) cells and immunosuppressive T regulatory (Treg) cells, contributing to systemic inflammation [3, 8].

According to the 2015 EULAR/ACR recommendations, standard therapy for PMR is oral glucocorticoid (GC) treatment with initial prednisolone doses of 12.5–25 mg, aiming for the lowest effective dose due to long-term side effects [9]. Off-label use of methotrexate (MTX) may be an option for PMR patients who suffer from GC-related toxicity or disease relapse although evidence is sparse [3]. Recent trials of IL-6 receptor inhibitors (tocilizumab and sarilumab) have shown superior efficacy over placebo, and new therapies are in development [10–14]. Sarilumab was recently approved by the FDA and the EMA for PMR patients with inadequate GC response or relapse [15, 16]. Patients treated with sarilumab had higher rates of sustained remission and lower cumulative GC doses compared with placebo [12]. Another monoclonal antibody, the IL-17A inhibitor secukinumab, is currently under phase 3 evaluation [13].

Current recommendations advise early confirmation of PMR, e.g. in fast-track clinics, to reduce misdiagnoses, prolonged GC therapy and hospital admissions [17–19]. However, there is still no standardized procedure of referring people with suspected PMR to rheumatologists, leading to disparities in treatment [17]. General practitioners (GPs) refer only ∼25% of potential PMR patients to rheumatologists. Furthermore, many patients already received GC treatment before being seen by a rheumatologist, and the referral-to-appointment time was long in general [20].

To improve the suggested implementations of the recommendations for early referral of PMR patients to specialist care, it is important to have up-to-date incidence rates of suspected cases [19]. The annual PMR incidence ranges from 113 in Norway to 13 per 100 000 per year in Italy, with a north–south gradient in Europe [3, 21, 22]. Previously reported incidence (17.7/100 000 people ≥40 years) and prevalence numbers (140–150/100 000 or 129.8/100 000 people) in Germany are low by international comparison [4, 23]. Based on these data, 66 000–71 000 PMR patients are currently estimated in Germany [23].

To our knowledge, there has been only one study that evaluated the incidence and prevalence of PMR in one federal state (Baden-Württemberg) of Germany [4]. In comparison with international data, this study showed a comparatively low PMR incidence and prevalence in a population ≥40 years. Thus, the primary aim of our database analysis was to provide updated and comprehensive population-based incidence and prevalence estimates of PMR in Germany between 2018 and 2021. Additionally, this study aimed to identify the groups of specialists in Germany who are responsible for the initial diagnosis of PMR and the initiation of treatment.

## Methods

### Database and ethics

The InGef (Institute for Health Research Berlin GmbH) database contains longitudinal anonymized healthcare claims data from ∼9 million insured members of >60 German statutory health insurance providers. Data across different healthcare sectors are available on patient level, and individuals can be followed over a period of up to 6 years. A sample of ∼4.8 million individuals per year was selected that was representative of the German population regarding age and sex, thus allowing us to study rare diseases or complex treatment patterns [24]. Since this study was based on anonymized claims data, ethical approval and informed consent of the patients was not required.

### Study design

The study aimed to analyse the epidemiology, patient characteristics and physician specialties involved in the diagnosis and treatment of PMR through a cross-sectional analysis using the InGef research database. The dataset covers all years from 2015 to 2022, while the prevalence and incidence were calculated for each year from 2018 to 2021. In addition, the calculation of the PMR incidence was based on considering a diagnosis-free baseline period of three calendar years prior to the respective study year. All persons who had already been diagnosed with PMR within this period were excluded from the study cohort for the incidence analysis to ensure that they were first-time or newly diagnosed cases.

The analyses to describe the received treatments for PMR were carried out longitudinally. A cohort was formed consisting of all previously identified patients with PMR between 2018 and 2020 and followed up during the 2 years after the incident diagnosis.

### Study populations

For the identification of prevalent patients, adults aged at least 50 years who were continuously insured in the InGef research database from 1 January of the respective study year until 31 December of the following year or until death were included in the study cohort. In addition, to identify incident patients, all persons had to be insured during the 3 years prior to the respective study year. Patients who had already developed PMR (according to the International Classification of Diseases and Related Health Problems, 10 edition, German Modification [ICD-10 GM] code M35.3) or GCA with PMR (ICD-10 GM M31.5) within 3 years prior to the respective study year were excluded (only for incident patients).

The additional study cohort for the description of prescribed medication included all incident patients between 2018 and 2020 who were fully observable for at least 2 years after their incident diagnosis or until death.

### Variables and measures

PMR patients were identified as such if at least one outpatient or inpatient main or secondary diagnosis ICD-10 GM code M35.3 was present in the respective study year. For additional validation of a first confirmed outpatient diagnosis, at least one further confirmed outpatient diagnosis, or an inpatient main or secondary diagnosis had to be present within three quarters after the index quarter (so called M2Q criterion). For patients with a first inpatient PMR diagnosis, the admission date of the respective hospital case was determined as the PMR index date. In the case of a first outpatient PMR diagnosis, the date of the first contact with the diagnosing physician (via the first billed code according to the uniform evaluation standard) was determined.

Patient characteristics include sex (female/male) and age. Age was defined as years on 1 January of the respective study year or at an index date (for incident patients only).

The main outcome is the 1-year prevalence and incidence of PMR according to the case definition of PMR patients. In addition, we reported predefined comorbidities of interest, defined by ICD-10 GM codes for inpatient or outpatient diagnoses. Data on comorbidities were gathered for each study year separately. Hence, not only pre-existing (before PMR diagnosis) but also newly diagnosed comorbidities were considered. Additionally, we evaluated predefined treatments, defined by the Anatomical Therapeutic Chemical (ATC) classification system, of which at least one prescription was filled in the respective year. As a further outcome, the specialist groups of the physicians who made the incident diagnosis were analysed. In addition, the groups of physicians who prescribed predefined medication to both prevalent and incident PMR patients were also analysed.

### Statistical analysis

Prevalence and incidence were reported as absolute and relative frequencies (per 100 000 persons) of eligible individuals at risk in the InGef database, with 95% CI assuming a binomial distribution. Additionally, age- and sex-standardized projections onto the total population in Germany were performed for the prevalence and incidence. For this projection, official population statistics from the German Federal Bureau of Statistics served as the reference [25]. This included calculating weighted averages of stratum-specific rates in the study population, using the corresponding numbers in each stratum of the standard German population (according to the German Federal Statistical Office) as weights. All other variables were analysed descriptively with absolute (*n*) and relative frequencies (%) and mean (SD) for age. The statistical analyses were performed using R statistical software (Version 4.0.2; R Foundation for Statistical Computing, Vienna, Austria).

## Results

The InGef database contained 4 266 888 people in the first study year (2018) and 4 321 057 people in the last study year (2021). Of these, 1 772 026 (2018) and 1 663 363 (2021) were fully observable and ≥50 years old and were therefore included in our observation.

### Prevalence and incidence of PMR

**Figure 1.**
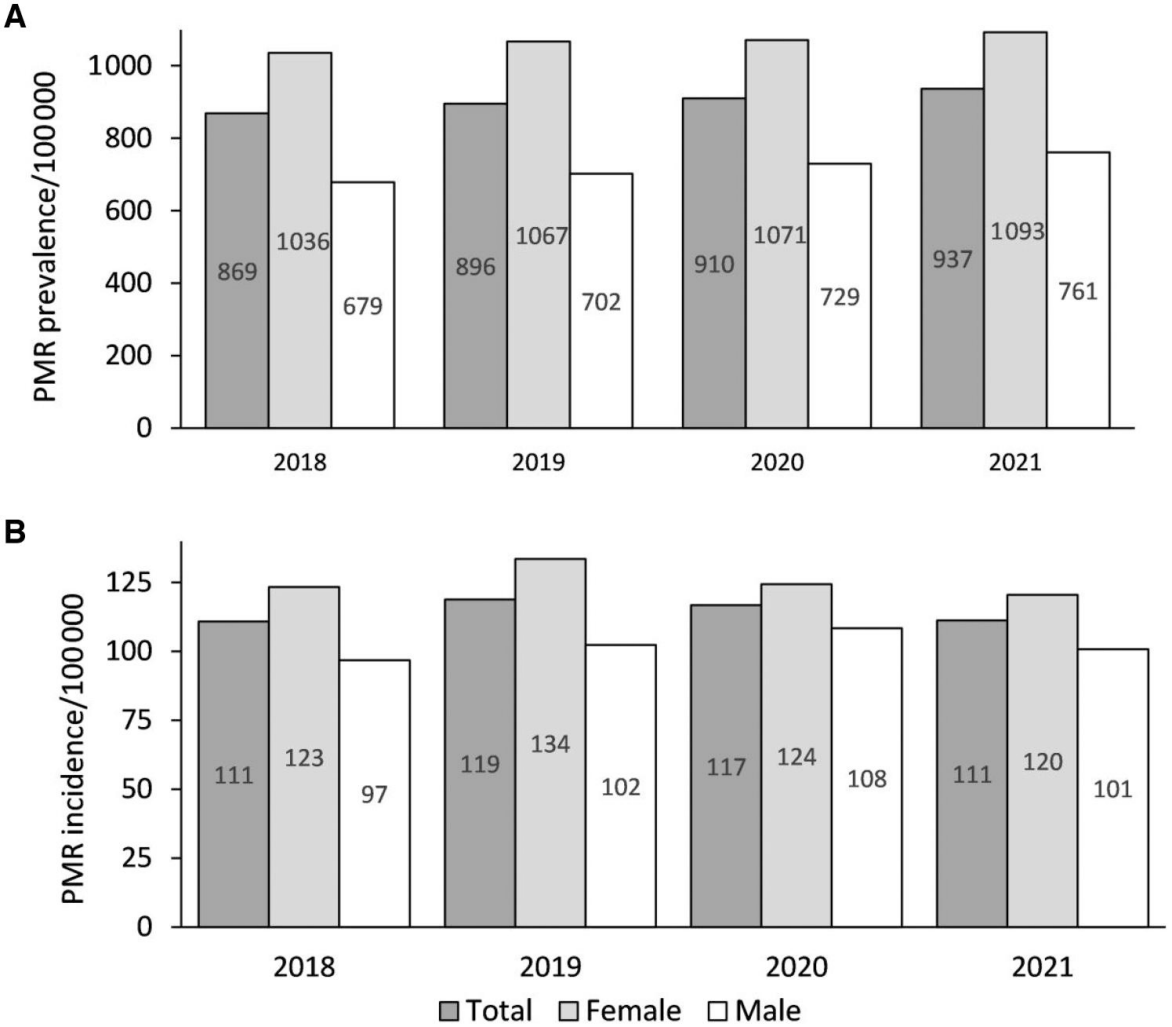
PMR prevalence (**A**) and incidence (**B**) in individuals aged ≥50 years in Germany stratified by sex. PMR: polymyalgia rheumatica

In the InGef cohort, on average 16 125 prevalent PMR patients at least 50 years of age were observed in each study year (2018: 15 767, 2021: 16 519; Supplementary Table S1, available at *Rheumatology* online). Our projection onto the total German population estimated 320 084 patients with PMR in 2018 and 351 340 patients in 2021. This amounts to a prevalence of 868.6 (95% CI: 865.6, 871.6) in the first study year (2018) and shows a slightly increasing trend to 936.9 (95% CI: 933.8, 939.9) per 100 000 people in Germany in the last study year (2021) (Fig. 1A). The prevalence of PMR was higher in women than in men across all years.

In the InGef cohort, on average 2004 newly diagnosed PMR patients at least 50 years of age were observed each study year (2018: 1986, 2021: 1908; Supplementary Table S1, available at *Rheumatology* online). Our projection onto the total German population estimated 40 386 newly diagnosed patients with PMR in 2018 and 41 206 patients in 2021. This amounts to an incidence of 110.8 (95% CI: 109.8, 111.9) in 2018 and shows a fluctuating course to 111.2 (95% CI: 110.1, 112.3) per 100 000 people in Germany in 2021 (Fig. 1B). The incidence of PMR was essentially higher in women than in men across all years.

### Patient characteristics

The prevalence showed a slight increase during the study period and peaked in the last year of the study, while the incidence showed a fluctuating trend; therefore, the following data refer to the last year, 2021. On average, prevalent patients were 75.7 (9.5) and incident patients were 72.1 (9.8) years of age in 2021 (Table 1, Supplementary Table S2, available at *Rheumatology* online). The majority of the prevalent (10 225, 61.9%) and incident (1100, 57.7%) patients were women.

**Table 1.** Demographic characteristics and relevant comorbidities in prevalent PMR patients

	2018	2019	2020	2021
	(*N* = 15 767)	(*N* = 16 224)	(*N* = 15 988)	(*N* = 16 519)
Age, mean (s.d.), years	75.0 (9.3)	75.2 (9.4)	75.5 (9.4)	75.7 (9.5)
Female, *n* (%)	9947 (63.1)	10 226 (63.0)	9965 (62.3)	10 225 (61.9)
Comorbidities (ICD-10-GM Code), *n* (%)				
Essential arterial hypertension (I10)	12 015 (76.2)	12 378 (76.3)	12 099 (75.7)	12 536 (75.9)
Disorders of lipoprotein metabolism and other lipidaemias (E78)	8621 (54.7)	8822 (54.4)	8739 (54.7)	9079 (55.0)
Diabetes mellitus (E10–E14)	4683 (29.7)	4822 (29.7)	4724 (29.5)	4904 (29.7)
Osteoporosis (M80/M81)	4452 (28.2)	4587 (28.3)	4391 (27.5)	4475 (27.1)
Cataract (H25/H26)	4201 (26.6)	4227 (26.1)	3841 (24.0)	4009 (24.3)
Chronic ischaemic heart disease (I25)	3733 (23.7)	3876 (23.9)	3787 (23.7)	3872 (23.4)
Chronic kidney disease (N18)	3054 (19.4)	3154 (19.4)	3084 (19.3)	3117 (19.2)
Glaucoma (H40)	1985 (12.6)	2079 (12.8)	1964 (12.3)	2049 (12.4)

ICD-10-GM: 10th revision of the International Classification of Diseases—German Modification; *N*: number of prevalent PMR patients in the InGef database; PMR: polymyalgia rheumatica.

The most common comorbidity in PMR prevalent patients was essential arterial hypertension. Cataract was observed in about one-fourth of the prevalent patients (Table 1).

### Diagnosing and treating physicians

The proportion diagnosed as inpatients was 13.3% (Table 2). Most frequently, outpatient diagnoses were made by general practitioners (37.1%), by internists in GP settings (22.2%), and by rheumatologists (11.4%) or orthopaedists (10.2%). After diagnosis, 21.9% of the PMR patients (with an available follow-up during the 2 years after the incident diagnosis) were referred to a rheumatologist.

**Table 2.** Specialty of diagnosing physician and referral to rheumatologist (InGef database)

	2018	2019	2020	2021
Total number of incident patients, *n* (%)	1986 (100)	2120 (100)	2001 (100)	1908 (100)
Inpatient index diagnosis, *n* (%)	239 (12.0)	256 (12.1)	228 (11.4)	254 (13.3)
Outpatient index diagnosis, *n* (%)	1747 (88.0)	1864 (87.9)	1773 (88.6)	1654 (86.7)
General practitioner	675 (38.6)	697 (37.4)	640 (36.1)	613 (37.1)
Internist general practice setting	396 (22.7)	413 (22.2)	366 (20.6)	368 (22.2)
Rheumatologist	174 (10.0)	217 (11.6)	240 (13.5)	189 (11.4)
Orthopaedist	169 (9.7)	189 (10.1)	173 (9.8)	168 (10.2)
Ophthalmologist	24 (1.4)	18 (1.0)	19 (1.1)	16 (1.0)
Other^a^	83 (4.8)	96 (5.2)	101 (5.7)	95 (5.7)
Not specified	226 (12.9)	234 (12.6)	234 (13.2)	205 (12.4)
Total number of incident patients with 2 years’ follow-up, *n* (%)	1802 (100)	1844 (100)	1760 (100)	—
Referral to rheumatologist^b^, *n* (%)	386 (21.4)	386 (20.9)	385 (21.9)	—

Only specialties that were given for >1% of the patients in all years are shown in detail. Specialties are expressed as percentage of the outpatient index diagnoses.

a Sum of other specialists (with <5 patients, respectively).

b Referral after diagnosis during follow-up period of 2 years. Since only data until 2022 were available, the follow-up period for patients diagnosed in 2021 was shorter than 2 years. Hence, the percentage of patients with a referral could not be calculated in 2021.

Within one study year, prevalent and incident patients were prescribed medications most frequently by general practitioners, rheumatologists and orthopaedists (Tables 3 and 4).

**Table 3.** Specialty of prescribing physician in prevalent patients (InGef database)

	2018	2019	2020	2021
	(*N* = 15 767)	(*N* = 16 224)	(*N* = 15 988)	(*N* = 16 519)
At least one prescription^a^, *n* (%)	9152 (58.0)	9295 (57.3)	8981 (56.2)	9218 (55.8)
General practitioner	4986 (31.6)	5021 (30.9)	4790 (30.0)	4881 (29.6)
Internist in general practice setting	3136 (19.9)	3194 (19.7)	3104 (19.4)	3121 (18.9)
Rheumatologist	2656 (16.8)	2799 (17.3)	2840 (17.8)	2996 (18.1)
Orthopaedist	748 (4.7)	729 (4.5)	751 (4.7)	739 (4.5)

a Predefined medication: glucocorticoids, methotrexat, IL-6 inhibitors (tocilizumab, sarilumab), Janus kinase inhibitors (tofacitinib, baricitinib, upadacitinib, filgotinib), conventional synthetic DMARDs (antirheumatic drugs and immunosuppressants), more than one prescription by different physicians per patient possible. *N*: number of prevalent patients in the InGef database.

**Table 4.** Specialty of prescribing physician in incident patients (InGef database)

	2018	2019	2020	2021
	(*N* = 1986)	(*N* = 2120)	(*N* = 2001)	(*N* = 1908)
At least one prescription^a^, *n* (%)	1659 (83.5)	1764 (83.2)	1706 (85.3)	1613 (84.5)
General practitioner	903 (45.5)	951 (44.9)	884 (44.2)	868 (45.5)
Internist in general practice setting	589 (29.7)	617 (29.1)	585 (29.2)	538 (28.2)
Rheumatologist	508 (25.6)	600 (28.3)	638 (31.9)	592 (31.0)
Orthopaedist	195 (9.8)	188 (8.9)	229 (11.4)	190 (10.0)

a Predefined medication: glucocorticoids, methotrexat, IL-6 inhibitors (tocilizumab, sarilumab), Janus kinase inhibitors (tofacitinib, baricitinib, upadacitinib, filgotinib), conventional synthetic DMARDs (antirheumatic drugs and immunosuppressants). *N*: number of incident patients in the InGef database.

### Prescribed medications after incident PMR diagnosis

To describe the medication received after a first PMR diagnosis, a total of 6027 incident PMR patients were analysed, within a 2-year follow-up period after their incident diagnosis.

The majority of these incident patients (89.4%) were treated at least once with one of the therapies for PMR (Table 5). Most frequently, GC and MTX were prescribed; 13.6% of the GC prescriptions were prescribed for <25 weeks, 43.3% for 25–52 weeks, 30.8% for >52–104 weeks, and 12.4% for >104 weeks (Supplementary Table S3, available at *Rheumatology* online). Alternative DMARDs such as sulfasalazine, leflunomide or azathioprine were prescribed much less frequently (<5% overall). Tocilizumab was prescribed in <2% of cases.

**Table 5.** Treatments in incident patients during the 2-year follow-up period (InGef population)

	2018	2019	2020
	(*N* = 1975)	(*N* = 2053)	(*N* = 1999)
At least one prescription, *n* (%)	1751 (88.7)	1803 (87.8)	1788 (89.4)
Glucocorticoid (H02AB)	1732 (87.7)	1787 (87.0)	1777 (88.9)
Methotrexate (M01CX01, L04AX03)	358 (18.1)	407 (19.8)	395 (19.8)
Tocilizumab (L04AC07)	36 (1.8)	28 (1.4)	30 (1.5)
Sulfasalazine, leflunomide, or azathioprine (M01CX02, L04AA13, L04AX01)	91 (4.6)	83 (4.0)	86 (4.3)

*N*: number of incident patients with follow-up period of 2 years in the InGef database.

## Discussion

In this projection of the InGef database on the German population, the prevalence of PMR was 869–937 per 100 000 residents aged 50 years and older, and the incidence was found to be between 111 and 119/100 000 during our study period (2018–2021). The prevalence of PMR increased by 7.8% (869–937/100 000) within the German population, whereas the incidence rates remained stable over the study years. Both incidence and prevalence were higher in women than in men. Most patients were initially diagnosed by GPs or an internist in a GP setting (in total 59.3%), followed by rheumatologists (11.4%). Treatment of PMR patients mainly comprised GC and to a lesser extent MTX. However, medication was only analysed from the first incident diagnosis onwards, so we cannot exclude that patients were already taking the medication before the incident diagnosis. On the other hand, PMR patients may receive GC treatment before a formal diagnosis was established, so prior or ongoing therapies may not be captured in our analysis. As a result, our reported medication rates and estimates of treatment duration and intensity may underestimate actual GC exposure. Essential hypertension, diabetes mellitus, osteoporosis and cataract were among the most frequent reported comorbidities in prevalent PMR patients.

Comparisons with data from different nations demonstrate that the incidence rates of PMR vary significantly depending on the research population’s country of origin. Similar incidences have been reported in Norway (112.6 per 100 000 population, 137.7 and 83.2 in women and men, respectively) and the UK (84–95.9/100 000) [21, 26, 27]. Slightly lower incidences were reported for Olmsted County, MN, USA (63.9/100 000) [28]. However, a previous study reported an incidence of only 17.7/100 000 in persons aged ≥40 years in Germany [4]. This is more in line with incidences in southern Europe, which range from 3.15 to 27.43/100 000 and Korea (2.06/100 000) [22, 29–33]. Accordingly, the prevalence in our cohort was 937/100 000, 1093 and 761 for women and men, respectively. This is significantly higher than previously reported German data with a prevalence rate of 129.8/100 000 (range 107.2–145.1/100 000) [4]. Prevalences in the USA (600–701/100 000), South America (200–255/100 000), Italy (370–620/100 000) and Korea (8.21/100 000) were also significantly lower [33–38]. On the other hand, higher PMR prevalence estimates were reported in the UK (2270/100 000) using GP records [39]. Importantly, the observed differences between countries might be caused amongst others by differing study designs, methodologies, definitions of PMR diagnosis and/or geographical coverages. For instance, one study only required PMR diagnosis to be recorded in the patient file, and at least two prescriptions for oral GC, whereas in other studies PMR patients had to meet specific diagnostic criteria [(i) age ≥50 years, (ii) bilateral pain and morning stiffness, and (iii) increased erythrocyte sedimentation rate], used physician billing data or hospitalization databases or health system databases and the ICD-10-GM code M35.3 [26, 30, 34, 36, 38, 40]. In our study, PMR cases were identified based on the ICD-10-GM code M35.3 as assigned by physicians according to clinical guidelines. We cannot rule out that some physicians did not validate their diagnosis based on additional clinical or laboratory data (in accordance with the EULAR/ACR recommendations). However, we only included PMR cases for which the physicians stated that the diagnosis was confirmed. Additionally, an outpatient PMR diagnosis in our study required at least one additional confirmed outpatient diagnosis or an inpatient primary or secondary diagnosis within three quarters following the index quarter. Hence, we assume that the risk of overestimating PMR cases in our study is limited.

Similar to our findings, another German study reported around 60% of diagnoses were performed by GPs, followed by rheumatologists or orthopaedists [4]. In our study 21.9% of those diagnosed with PMR were referred to a rheumatologist after diagnosis, which is in line with an international survey regarding PMR management practices [20]. However, a study in the UK reported that 44.4% of PMR patients are referred for secondary care for review [41]. According to the German guidelines most patients were treated with GC, followed by MTX [42]. However, information on reasons for prescription of GC or MTX was not available. Hence, it cannot be ruled out that these therapies were prescribed for other (non-chronic) indications.

In our study, essential hypertension and disorders of lipoprotein metabolism, which are cardiovascular disease risk factors, as well as chronic ischaemic heart disease were among the highest ranked comorbidities. Hypertension was present in 75% of PMR patients, exceeding the 60% prevalence observed in the German population >65 years of age [43]. Similar to our findings, a Japanese study reported hypertension, diabetes mellitus and osteoporosis in >25% of PMR patients [44]. Several studies (reviewed in [45]) reported that patients with PMR had vascular diseases significantly more often than control patients. It has been suggested that PMR patients are susceptible to develop atherosclerosis. Through a number of different molecular pathways, systemic inflammation can contribute to endothelial dysfunction and/or atherosclerosis [46]. Some of the reported comorbidities in the prevalent population of our study, such as arterial hypertension, diabetes mellitus, osteoporosis and cataract, may be linked to GC treatment [46, 47]. In our study, comorbidities could be pre-existing (before PMR diagnosis) or newly developed in the respective study year.

Although the analysis dataset obtained from the InGef database covers ∼4.8 million insured members of social health insurances all over Germany, representativeness for the whole population can only be guaranteed with regard to age and sex. Data might not be representative for other demographic or clinical characteristics, such as urban and rural distribution, socioeconomic status or access to specialist care. This limitation could introduce selection bias, with certain subpopulations potentially being under- or over-represented. As the InGef data base showed good accordance with German reference data regarding hospitalization and overall mortality rates [24], we assume that also the PMR incidences and prevalences in the InGef database are representative for the German population.

Furthermore, the analysis might be subject to the immeasurable time bias, as medications during a hospital stay cannot be identified in German claims data. In addition to the billing information relevant to a treatment case used in this analysis, no further information such as individual patient records or disease-specific laboratory values can be undertaken to validate specific clinical pictures or the disease severity of patients in detail. The last two years of the study period were during the COVID pandemic. We can only speculate how this had an impact on the diagnosis, referral and overall management of patients with PMR. In this study, PMR diagnoses were often made by GPs and were not verified by a second diagnosis. Hence, the number of PMR cases might be inflated due to misdiagnoses.

In conclusion, PMR was more frequent in this analysis in patients aged 50 years and older than previously assumed. The differential diagnosis of PMR is challenging as subclinical GCA or rheumatoid arthritis may be present, requiring imaging such as ultrasound and the clinical experience of a rheumatologist. As the initial correct diagnosis influences prognosis and treatment, rheumatologists should be involved earlier and more frequently in the care of PMR patients.

## Supplementary Material

keaf367_Supplementary_Data

## Data Availability

The data underlying this article cannot be shared publicly for reasons of privacy of individuals that participated in the study.
