# Supplementary material for: Frequency, diagnosis, and management of polymyalgia rheumatica in Germany—database analysis of medical insurance data
Source: Rheumatology (Oxford). 2025 Jul 7;64(11):5818–25. doi: 10.1093/rheumatology/keaf367 (PMC12598749; doi:10.1093/rheumatology/keaf367)
Supplement: keaf367_Supplementary_Data [file keaf367_supplementary_data.docx]

**SUPPLEMENTAL MATERIAL**

**Frequency, Diagnosis, and Management of Polymyalgia Rheumatica in Germany – Database Analysis of Medical Insurance Data**

**Table S1**. Number of prevalent and incident PMR patients in the InGef population

|  | **Prevalence of PMR** | | | **Incidence of PMR** | | |
| --- | --- | --- | --- | --- | --- | --- |
| **Year** | **Prevalent patients** | **Insured persons** | **per 100,000** | **Incident patients** | **Insured persons** | **per 100,000** |
| 2018 | 15,767 | 1,772,026 | 889.77 | 1,986 | 1,751,640 | 113.38 |
| 2019 | 16,224 | 1,757,820 | 922.96 | 2,120 | 1,737,088 | 122.04 |
| 2020 | 15,988 | 1,663,582 | 961.06 | 2,001 | 1,642,918 | 121.80 |
| 2021 | 16,519 | 1,663,363 | 993.11 | 1,908 | 1,642,156 | 116.19 |
| PMR: polymyalgia rheumatica | | | | | | |

**Table S2**. Demographics

|  | **2018**  (N=1,986) | **2019**  (N=2,120) | **2020**  (N=2,001) | **2021**  (N=1,908) |
| --- | --- | --- | --- | --- |
| Age (years), mean ± SD | 71.5 ± 9.7 | 71.7 ± 9.6 | 72.1 ± 9.7 | 72.1 ± 9.8 |
| Female, n (%) | 1,169 (58.9) | 1,265 (59.7) | 1,133 (56.6) | 1,100 (57.7) |
| PMR: polymyalgia rheumatica, N: Number of incident PMR patients in the InGef database, SD: Standard deviation | | | | |

**Table S3.** Estimated length of intake of glucocorticoids within the follow-up period (8 quarters) in incident patients (InGef population)

|  | **2018** | **2019** | **2020** |
| --- | --- | --- | --- |
|  | n (%) | n (%) | n (%) |
| Incident patients with a second consecutive prescription of GC | 1,430 (100.0) | 1,512 (100.0) | 1,476 (100.0) |
| ≤ 25 weeks | 184 (12.9) | 193 (12.8) | 200 (13.6) |
| >25 - ≤ 52 weeks | 623(43.6) | 642 (42.5) | 639 (43.3) |
| > 52 - ≤ 104 weeks | 418(29.2) | 485 (32.1) | 454 (30.8) |
| > 104 weeks | 205 (14.3) | 192 (12.7) | 183 (12.4) |
